# Bladder irrigation with tap water to reduce antibiotic treatment for catheter-associated urinary tract infections: an evaluation of clinical practice

**DOI:** 10.3389/fruro.2023.1172271

**Published:** 2023-04-27

**Authors:** Felice E. E. van Veen, Stefan Den Hoedt, Rosa L. Coolen, Jessica Boekhorst, Jeroen R. Scheepe, Bertil F. M. Blok

**Affiliations:** Department of Urology, Erasmus Medical Center, Rotterdam, Netherlands

**Keywords:** urinary catheterization, intermittent urethral catheterization, catheter-associated urinary tract infections (CAUTI), antibiotics, antibiotic resistance, tap water, bladder irrigation, neurogenic lower urinary tract dysfunction (NLUTD)

## Abstract

**Introduction:**

Catheter-associated urinary tract infection (CAUTI) is a common complication among patients with urinary catheters and is often treated with antibiotics. With increasing rates of antibiotic resistance, it is necessary to explore alternative treatment options for CAUTIs. The aims of this study were 1) to assess the efficacy and treatment satisfaction of bladder irrigation (BI) with tap water to prevent and treat CAUTIs, 2) and to evaluate the current use of BI for CAUTIs among Dutch clinicians.

**Methods:**

The first part of this study consisted of a cross-sectional study among patients with intermittent or indwelling catheters who performed BI with tap water between March 2020 and May 2021. Efficacy, treatment satisfaction, and Patient Global Impression of Improvement (PGI-I) were assessed using questionnaires. Outcomes were compared between neurogenic lower urinary tract dysfunction (NLUTD) and non-NLUTD patients. Factors associated with overall treatment satisfaction were determined using logistic regression analysis. Second, a nationwide survey of Dutch clinicians was conducted to evaluate the current use of BI for CAUTIs.

**Results:**

A total of 99 patients who were performing BI for at least three months were included. The median age was 61.9 years, 41.4% had NLUTD, and 72.2% performed BI >1 year. The majority of both NLUTD (65.9%) and non-NLUTD patients (68.4%) were (very) satisfied with BI. Women had higher odds of reporting higher satisfaction and each additional CAUTI decreased the odds. Most NLUTD (85.4%) and non-NLUTD (65.5%) patients reported an improvement on the PGI-I with a difference in favour of NLUTD patients (p=0.002). In addition, 40.4% of the patients had no CAUTI, and 59.6% reported 1.39 (SD 2.06) CAUTIs. Only half of these self-reported CAUTIs were treated with antibiotics. In addition, 33 (58.9%) clinicians used BI for CAUTIs, of which ten used tap water as irrigation agent.

**Discussion:**

This study provides first evidence supporting the efficacy of BI with tap water in the treatment of CAUTIs and reducing the use of antibiotics. Patients are overall satisfied and experience improvement in their condition with BI. In addition, the majority of the surveyed Dutch clinicians use BI for CAUTIs. However, irrigation with tap water is still not widely used.

## 1 Introduction

Catheter-associated urinary tract infection (CAUTI) is a common complication among patients with an indwelling urinary catheter. CAUTIs also occur in patients performing clean intermittent catheterization (CIC), which is the treatment of choice in urinary retention (1). Especially with long-term usage of catheters the incidence rate of CAUTIs is high, ranging from 3.1 to 7.5 CAUTIs per 1000 catheter days (2). In addition, the daily risk of acquiring a CAUTI is estimated at 5%, depending on diagnostic criteria, study design and catheter type (3). With more than 100.000 patients using urinary catheters in the community setting in the Netherlands alone (4), CAUTIs are a source of a substantial healthcare burden.

CAUTIs are often treated with antimicrobial therapy after obtaining a urine specimen for culture according to the recommendations of the European Association of Urology (EAU) Guidelines of Urological Infections (5). Overuse of antibiotic treatment for urinary tract infections has contributed to the growing problem of antibiotic resistance among bacterial uropathogens (6, 7). Patients with urinary catheters often receive antibiotics without urgent need, as bacteriuria frequently occurs with no or mild symptoms, such as cloudy or strong-smelling urine. As a consequence, these patients have an increased likelihood of having CAUTIs with antimicrobial resistant bacteria (8), such as extended spectrum beta-lactamase (ESBL) producing Enterobacteriaceae (9). Antibiotic resistance is a globally recognized healthcare threat, causing prolonged hospitalizations, increased mortality and leading to higher medical costs (10). These consequences emphasize the importance of being cautious with the use of antibiotics. Therefore, we think it is critically important to explore alternatives for antibiotic treatment of CAUTIs with no symptoms of tissue invasion.

Urinary bladder irrigation (BI) is commonly performed as standard management of long-term urinary catheters (11), but it remains a controversial method to reduce CAUTIs. A Cochrane review found inconclusive evidence for the role of BI in preventing CAUTIs (12). On the other hand, two additional studies provided evidence supporting the effectiveness of BI with sterile saline in preventing CAUTIs in critically ill comatose patients and children catheterized after bladder surgery (13, 14). At our institute, the Erasmus Medical Center (Erasmus MC), we use tap water for BI as current practice, without evidence of an increased risk of CAUTIs. Taking into account that tap water in the Netherlands is of high quality and is continuously monitored for microbiological agents (15, 16). Moreover, in two previous studies, tap water was used as irrigation solution to prevent bladder calculi in augmented bladders and to dilute solution G for BI without resulting in an increased risk of CAUTIs (17, 18). In most countries tap or drinking water is readily available and is therefore easy to implement for patients, providing a more cost-effective alternative. No previous studies have investigated BI with tap water for the prevention and treatment of CAUTIs. Therefore, we aimed to assess the safety, efficacy and treatment satisfaction of BI with tap water to prevent and treat CAUTIs with no symptoms of tissue invasion among neurogenic lower urinary tract dysfunction (NLUTD) and non-NLUTD patients. In addition, we aimed to evaluate the current use of BI, including tap water, as a treatment for CAUTIs among clinicians in the Netherlands.

We hypothesized that BI with tap water can reduce the use of antibiotics in the prevention and treatment of CAUTIs, and that patients are overall satisfied with this treatment. The rationale behind this hypothesis is that BI with tap water could theoretically reduce bacterial overgrowth in the urinary bladder and prevent catheter blockage by washing out bacterial overgrowth and debris. Another potential mechanism of action of BI with tap water is its hypo-osmolarity. The exposure of a hypo-osmolar medium to microorganisms, such as E. Coli, results in loss of viability and lysis of the microbes (19). This could theoretically reduce the risk of bacteriuria and CAUTIs. Furthermore, we expect a relatively low use of BI among clinicians, since BI is not recommended by the guidelines of the EAU or American Urological Association (AUA).

## 2 Materials and methods

### 2.1 Study design and population

Ethical approval was granted by the Institutional Review Board of the Erasmus MC, Rotterdam, the Netherlands (MEC-2021-0855). The first part of this study consists of a cross-sectional study that was conducted between February and April 2022 at the Erasmus MC. Patients 18 years or older who performed BI between October 2019 and November 2021 were identified from the electronic health record (EHC). Patients were included when they had a transurethral catheter (TUC), suprapubic catheter (SPC) or performed clean intermittent catheterization (CIC) and performed BI with tap water during the last three months before inclusion. All eligible patients received an informed consent form and online questionnaire by email. In case of no response, a weekly reminder up to two weeks was sent by email. Hereafter, patients were contacted by telephone to ask for their participation. Patients who were unable to complete the questionnaire online, received a paper-based questionnaire by mail or completed the questionnaire over the phone.

Patients’ medical charts were reviewed to compile the following data: sex, age, type of urinary catheter, etiology of catheterization (NLUTD or non-NLUTD), underlying neurogenic etiology, treatment for (non-)NLUTD (antimuscarinic use, mirabegron use, intradetrusor OnabotulinumtoxinA (BoNT-A), Sacral neuromodulation (SNM)), history of ileocystoplasty and (catheterisable) urinary stoma, antibiotic prophylaxis use, immunosuppressant use, start date of BI and the indication for BI.

### 2.2 Questionnaire

All included patients received a questionnaire to assess the efficacy, Patient Global Impression of Improvement (PGI-I) (20) and treatment satisfaction. The efficacy was determined with the number of self-reported CAUTIs, antibiotic treatment for CAUTIs and CAUTI-associated hospitalizations in the past three months before completing the questionnaire. The PGI-I was used to assess the improvement in the patient’s condition or situation due to BI. Additionally, treatment satisfaction was measured using a five-point Likert scale on four items including overall satisfaction, acceptability of BI frequency, acceptability of BI duration and treatment recommendation to other patients. Patients were asked if BI had a positive and/or negative impact on their lives and, if so, for what reasons (multiple reasons could be given).

### 2.3 Bladder irrigation

BI with tap water was prescribed to patients with recurrent CAUTIs as a primary preventive measure or as treatment for CAUTIs without symptoms of tissue invasion including fever, flank pain and delirium. CAUTI symptoms include cloudy or strong-smelling urine, haematuria, dysuria/pain during catheterization, urinary frequency, urinary urgency and suprapubic pain. BI with tap water was also prescribed for other catheter-related problems, including catheter encrustation, preventing bladder stones and severe debris or mucous production (e.g., with ileocystoplasty or neobladder) to prevent catheter blockage and CAUTIs. Bladder stones were excluded *via* ultrasound prior to the start of BI.

All patients received instructions from the continence nurses to perform BI. These consisted of actively injecting and aspirating 50 mL of tap water into the bladder with a catheter-tip syringe until the effluent was clear. Tap water at body temperature was preferred to prevent bladder cramps during BI. Patients used a phase-out schedule starting with daily BI in the first week followed by: every other day, twice a week, once a week and hereafter stop BI. They were instructed to phase out when the effluent of the first syringe was clear. Daily BI was resumed when patients were experiencing symptoms of a CAUTI or severe debris/mucous production. In addition, some patients continued BI daily to prevent CAUTIs. Patients were instructed to contact their physician and discontinue BI in the presence of fever or other symptoms of tissue invasion. Patients could receive antibiotic prophylaxis in addition to BI. Other therapies for CAUTIs such as cranberries, phytotherapy and acidation with solution G and R were not prescribed.

### 2.4 Clinician survey

The second part of the study consists of a nationwide survey among Dutch urologists and urology residents to evaluate the current implementation of BI with tap water for the treatment of CAUTIs. A short survey with four questions investigated the use of BI as treatment for CAUTIs and the use of tap water as irrigation solution. The survey was administered to 86 members of the functional urology focus group of the Dutch Urological Association.

### 2.5 Statistical analysis

Baseline characteristics and outcome variables of the questionnaire were presented as descriptive statistics and were compared between NLUTD and non-NLTUD patients. The normality of distribution was assessed using the Shapiro-Wilk W test. Differences between NLUTD and non-NLUTD patients were evaluated using the Chi-square test for unordered categorical data, the Chi-square test or Fisher’s exact test for binary data, and Mann-Whitney U-test for ordinal data and non-normally distributed continuous data. A p-value <0.05 was considered statistically significant and all tests were two sided.

A multivariable ordinal logistic regression analysis with the proportional odds model was performed to determine variables associated with overall treatment satisfaction of BI. Sex, age, catheter type, etiology of catheterization, number of self-reported UTIs and frequency of BI were entered in the model. Missing data were handled with multiple imputation by chained equations, assuming missing at random (21). Outcomes of the clinician survey were presented as descriptive statistics. The statistical analyses were performed in R version 4.1.2., and R package mice version 3.14.0 was used for multiple imputation (22).

## 3 Results

**Figure 1:**
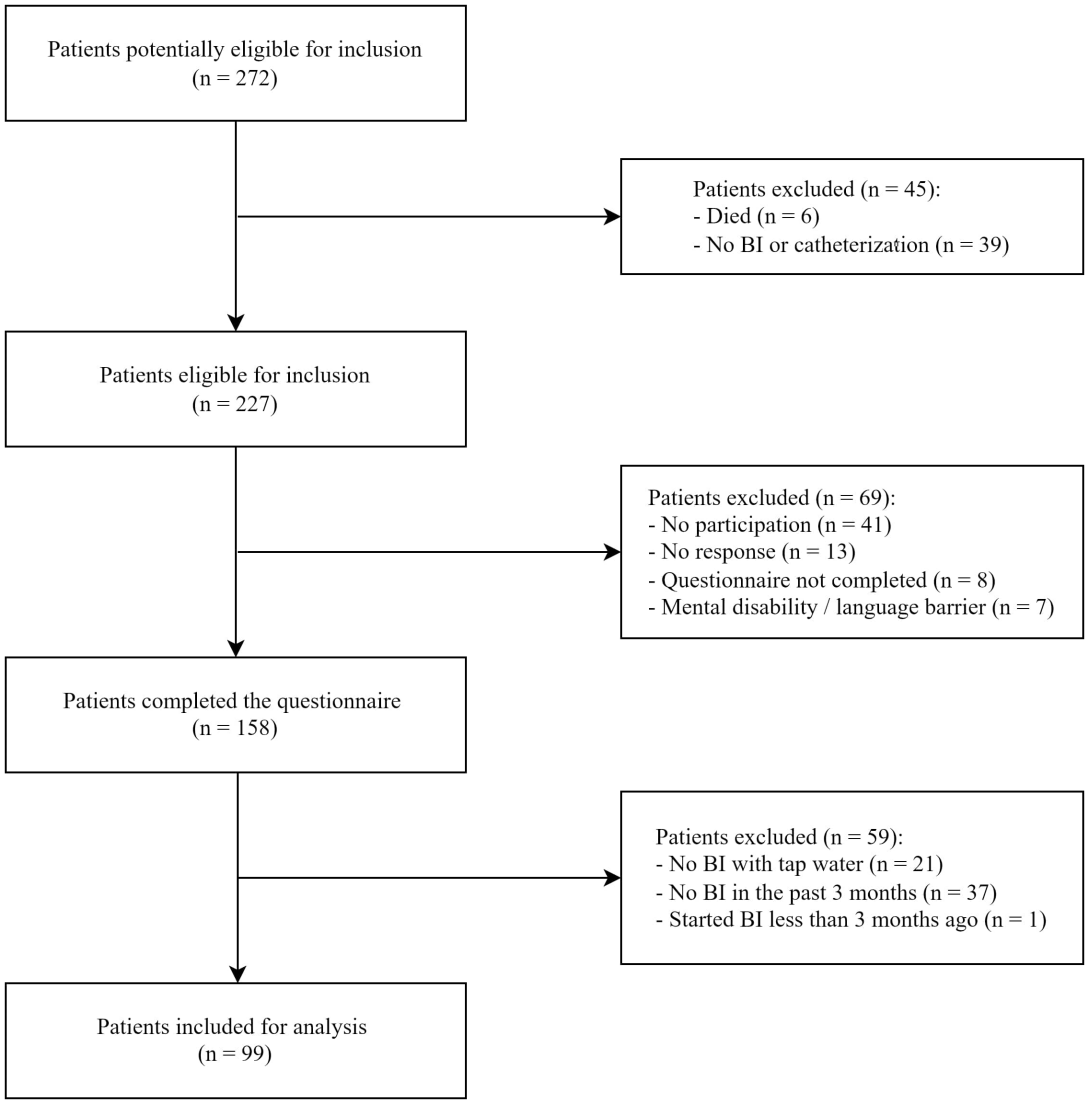
Flowchart of patient inclusion.

A total of 272 patients were identified from the EHC, of whom 227 patients were eligible for inclusion. Of these 227 patients, 159 (70.0%) patients completed the questionnaire of which 59 were excluded due to: no BI with tap water (n = 21), no BI in the past three months (n = 37), and less than three months of experience with BI (n = 1). A total of 99 patients who performed BI with tap water at least three months prior to completion of the questionnaire were included in this study (**Figure 1**). The patient characteristics are listed in **Table 1**. The median age at completion of the questionnaire was 61.9 years (IQR 71.0-44.7). The majority of the patients were on CIC (64.6%), had more than one year of experience with BI (72.7%) and had CAUTIs as indication for starting with BI (66.7%). Antibiotic prophylaxis was used by 14 (14.1%) patients in addition to BI. Within this cohort, 41 (41.4%) patients had a neurogenic cause as underlying disease for catheterization. NLUTD patients were younger (49.0 vs 65.5 years), were more likely to have an SPC (26.8% vs 6.9%) and to use mirabegron (14.6% vs 1.7%) compared to non-NLUTD patients. Of all patients, 7 (7.1%) used mirabegron, 19 (19.2%) used antimuscarinics, 23 (23.2%) received BoNT-A, and 8 (8.1%) had SNM.

**Table 1:** Baseline characteristics.

Parameter	Total (n = 99)	NLUTD (n = 41)	Non-NLUTD (n = 58)	P value
Sex (%)				0.577
Women	62 (62.6)	27 (65.9)	35 (60.3)	
Men	37 (37.4)	14 (34.1)	23 (39.7)	
Age (median [IQR])	61.00 [71.00-44.00]	49.00 [32.50, 63.5]	65.50 [55.50, 73.25]	<0.001
Neurogenic cause (%)				
Spinal cord injury/transverse myelitis	–	19 (46.3)	–	
Multiple sclerosis	–	6 (14.6)	–	
Spina bifida	–	4 (9.8)	–	
Others	–	12 (29.2)	–	
Catheter type (%)				0.022
CIC	64 (64.6)	22 (53.7)	42 (72.4)	
TUC	20 (20.2)	8 (19.5)	12 (20.7)	
SPC	15 (15.2)	11 (26.8)	4 (6.9)	
CIC frequency/day (median[IQR])°	5.0 [6.0-3.0]			
(Catheterisable) Urinary stoma (%)	24 (24.2)	10 (24.4)	14 (24.1)	0.977
Ileocystoplasty (%)	8 (8.1)	5 (12.2)	3 (5.2)	0.270
Antibiotic prophylaxis use (%)	14 (14.1)	6 (14.6)	8 (13.8)	0.906
Immunosuppressants use (%)	6 (6.1)	3 (7.3)	3 (5.2)	0.690
Mirabegron use (%)	7 (7.1)	6 (14.6)	1 (1.7)	0.014
Antimuscarinic use (%)	19 (19.2)	9 (22.0)	10 (17.2)	0.369
Intradetrusor BoNT-A (%)	23 (23.2)	13 (9.5)	10 (13.5)	0.146
SNM (%)	8 (8.1)	0 (0.0)	8 (19.5)	0.011
Time since start BI (%)				0.156
3-12 months	27 (27.3)	9 (22.0)	18 (31.0)	
1-3 years	54 (54.5)	22 (53.7)	32 (55.2)	
>3 years	18 (18.2)	10 (24.4)	8 (13.8)	
Indication for BI (%)*				0.381
CAUTI	67 (67.7)	27 (65.9)	43 (75.1)	
Debris/mucous production	36 (36.4)	17 (41.5)	16 (27.6)	
Prevention encrustation/bladder stones	16 (16.2)	9 (22.0)	7 (12.1)	
Frequency of BI (%)				0.361
If necessary	31 (31.3)	13 (31.7)	18 (31.0)	
Daily (%)	43 (43.4)	21 (51.2)	22 (37.9)	
Number daily (mean (SD))	2.00 (2.36)	1.67 (1.11)	2.32 (3.12)	
Weekly (%)	25 (25.2)	7 (17.1)	18 (31.0)	
Number weekly (mean (SD))	1.68 (0.80)	1.71 (0.95)	1.67 (0.77)	
Duration of BI (%)				0.856
0 - 15 minutes	86 (86.9)	36 (87.8)	50 (86.2)	
15 - 30 minutes	12 (12.1)	4 (9.8)	8 (13.8)	
30 minutes - 1 hour	1 (1.0)	1 (2.4)	0 (0.0)	
Questionnaire completion (%)				0.437
E-mail	91 (91.9)	39 (95.1)	52 (89.7)	
Phone	2 (2.0)	1 (2.4)	1 (1.7)	
Post	6 (6.1)	1 (2.4)	5 (8.6)	

NLUTD, neurogenic lower urinary tract dysfunction; CIC, clean intermittent catheterization; TUC, transurethral catheter; SPC, suprapubic catheter; BI, bladder irrigation; BoNT-A, OnabotulinumtoxinA. SNM, Sacral neuromodulation. *Patients could have multiple indications for BI. °n =64.

### 3.1 Safety and efficacy

During the last three months of BI, 40.4% (40/99) of the patients had no CAUTI, and 59.6% (59/99) experienced CAUTI symptoms with an average of 1.39 times (SD2.06) (**Table 2**). Of all patients who had complaints of a CAUTI, only 57% (34/59) needed antibiotic treatment for a CAUTI. Furthermore, a mean number of 0.50 antibiotic treatments per CAUTI was observed in the patients who experienced CAUTI symptoms. This indicates that only half of all self-reported CAUTIs were treated with antibiotics. Three (3%) patients were hospitalized because of a CAUTI. There were no significant differences in the number of self-reported CAUTIs, antibiotic treatments and hospitalisations between NLUTD and non-NLUTD patients.

**Table 2:** Safety and efficacy of bladder irrigation with tap water.

Parameter	Total (n = 99)	NLUTD (n = 41)	Non-NLUTD (n = 58)	P value
Number of CAUTIs (%)*				0.885
0	40 (41.7)	18 (45.0)	22 (39.3)	
1	26 (27.1)	9 (22.5)	17 (30.4)	
2	12 (12.5)	6 (15.0)	6 (10.7)	
≥3	18 (18.8)	7 (17.5)	11 (19.6)	
Number of AB treatments for CAUTIs (%)°				0.598
0	64 (65.3)	28 (68.3)	36 (63.2)	
1	25 (25.5)	8 (19.5)	17 (29.8)	
2	5 (5.1)	3 (7.3)	2 (3.5)	
≥3	4 (4.1)	2 (4.9)	2 (3.5)	
Ratio AB treatment: CAUTI (mean(SD))*	0.50 (0.48)	0.49 (0.47)	0.51 (0.49)	0.935
Hospitalisations (%)	3 (3.0)	1 (2.4)	2 (3.4)	1.000

NLUTD, neurogenic lower urinary tract dysfunction; CAUTI, catheter-associated urinary tract infection; AB, antibiotic. *Missing value for 1 NLUTD and 2 non-NLUTD patients. °Missing value for 1 non-NLUTD patient.

### 3.2 Treatment satisfaction and PGI-I

**Figure 2:**
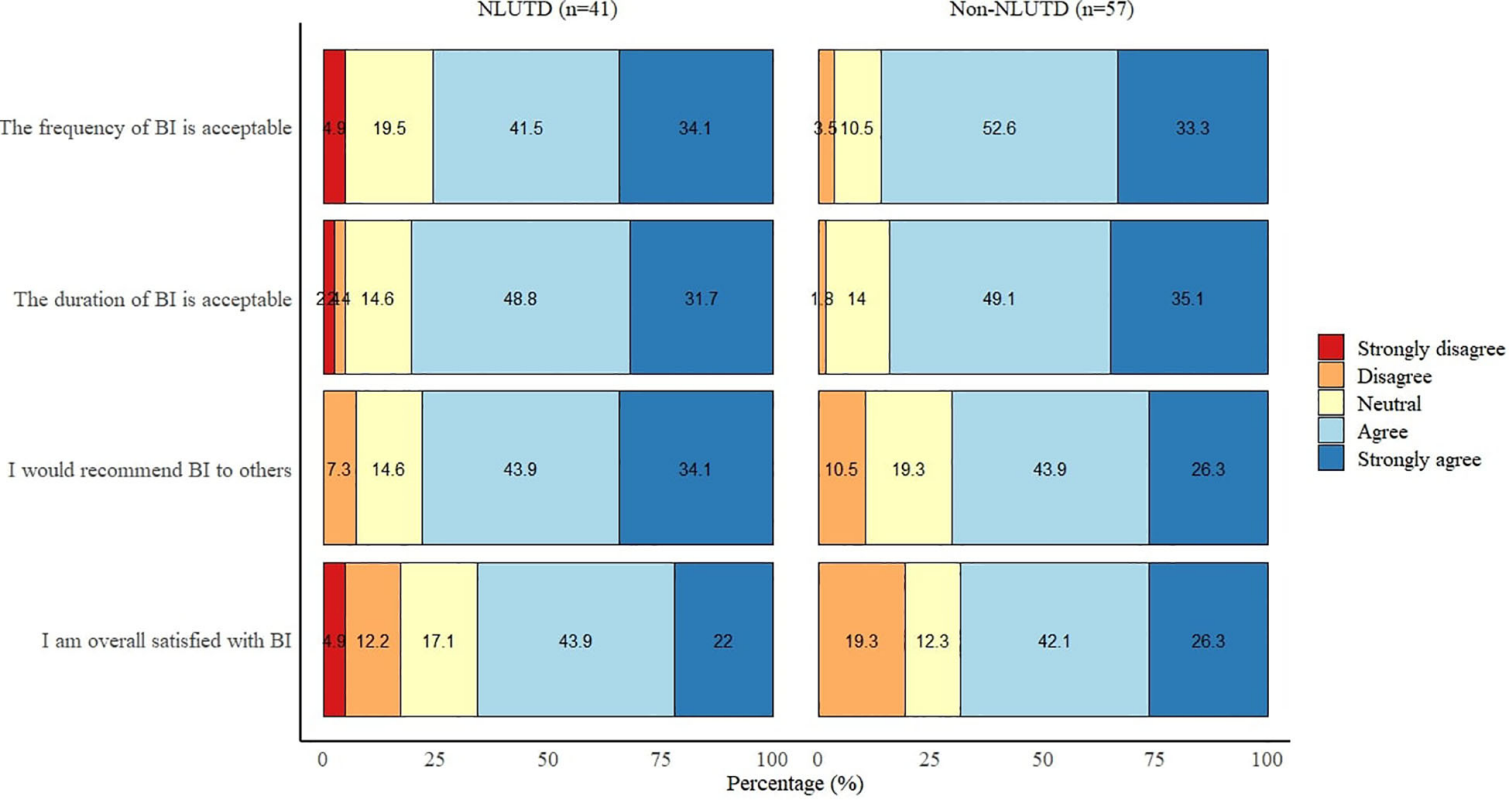
Treatment satisfaction outcomes of bladder irrigation with tap water.

The majority of both NLUTD (65.9%, n=27) and non-NLUTD patients (68.4%, n=39) were overall satisfied or very satisfied with BI. This is in contrast to seven (17.1%) NLUTD and 11 (19.3%) non-NLUTD patients who were not satisfied with BI. The majority of all patients found the duration (81.8%, n=81) and frequency (79.8%, n=79) of BI acceptable and would recommend BI to other patients (73.7%, n=73) (**Figure 2**). There were no significant differences between NLUTD and non-NLUTD patients on all four outcomes of treatment satisfaction. In addition, BI had a positive impact on life in 54 patients (54.5%), due to the following reasons: fewer CAUTI symptoms (n = 49), fewer catheter obstructions (n = 3), and fewer abdominal pain or bladder spasms (n = 2). BI had a negative impact on life in 21 patients (21.2%), due to the following reasons: makes them dependent on others (n = 10), makes life difficult (n = 7), are concerned that BI causes more complaints (n = 3), BI is painful (n = 3), and it takes too much time (n = 2).

A multivariable ordinal logistic regression analysis showed that sex and the number of self-reported CAUTIs were significantly associated with overall treatment satisfaction (**Table 3**). After multiple imputation and adjusting for confounders, female patients had 2.38 higher odds (95% CI: 1.07-5.30, p=0.037) of reporting a higher satisfaction compared with male patients when holding all other predictor variables constant. For the number of CAUTIs, each additional self-reported CAUTI decreased the odds of higher satisfaction 1.40 times (OR 0.73, 95% CI: 0.57-0.93; p=0.012).

**Table 3:** Multivariable ordinal logistic regression analysis of factors associated with overall treatment satisfaction.

Parameter	OR (95% CI)	P-value
Age (y)	1.00 (0.97 to 1.03)	0.946
Sex (reference = man)		
Woman	2.38 (1.07 to 5.30)	0.037
Catheter type (reference = CIC)		
TUC	0.92 (0.32 to 2.66)	0.885
SPC	2.77 (0.81 to 9.50)	0.109
Self-reported CAUTIs (n)	0.73 (0.57 to 0.93)	0.012
Neurogenic bladder (reference = no)		
Yes	1.49 (0.61 to 3.65)	0.389
Frequency (reference = if necessary)		
Daily	0.63 (0.25 to 1.62)	0.341
Weekly	1.05 (0.36 to 3.02)	0.932

CIC, clean intermittent catheterization; TUC, transurethral catheter; SPC, suprapubic catheter; CAUTI, catheter-associated urinary tract infection.

**Figure 3:**
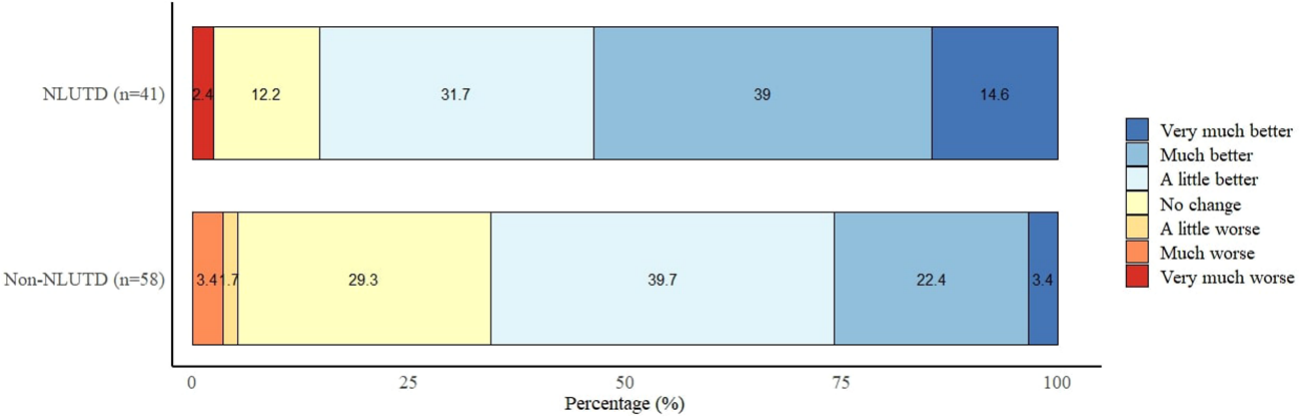
PGI-I outcomes of bladder irrigation with tap water.

**Figure 3** shows the PGI-I outcomes. Among NLUTD patients, 85.4% (35/41) reported an improvement in their condition after starting with BI. The reported improvement on the PGI-I scale was significantly different from those with non-NLUTD (p=0.002). Of those patients, 65.5% (38/58) reported having an improvement in their condition after starting with BI.

### 3.3 Clinician survey

A total of 56/86 clinicians from 32/82 different hospitals completed the short survey including 42 urologists, 12 urology residents and two with unknown clinician status (**Table 4**). Thirty-three (58.9%) clinicians reported that they use BI as a treatment for CAUTIs in addition to or instead of oral antibiotics. These clinicians used different solutions for BI: sodium chloride (66.7%), solutio R/G (51.5%), gentamycin (27.3%) and povidone iodine (9.1%). Only 10 (30.3%) clinicians used tap water as bladder irrigation agent.

**Table 4:** Clinician survey.

Parameter	Total (n = 56)
Clinician status (%)	
Urologist	42 (75.0)
Urology resident	12 (21.4)
Unknown	2 (3.6)
Do you ever initiate BI in patients with a CAUTI in addition to or instead of oral antibiotics? (%)	
Yes	33 (58.9)
No	23 (41.1)
Which BI agent do you use for this treatment? (%)*	
Sodium chloride	22 (66.7)
Solutio R/G	17 (51.5)
Gentamycin	9 (27.3)
Tap water	10 (30.3)
Povidone iodine	3 (9.1)

BI, bladder irrigation; CAUTI, catheter-associated urinary tract infection.* n = 33, multiple answers possible.

## 4 Discussion

Patients with indwelling urinary catheters or performing CIC have an increased risk of developing CAUTIs (23). CAUTIs occur due to uropathogens entering the urinary tract through urinary catheters. Nearly 100% of patients with urinary catheters will have bacteriuria (24), which can become a CAUTI when causing symptoms. CAUTIs have been associated with prolonged hospitalization, increased morbidity, mortality and healthcare costs (25). Generally, antibiotics are required to counteract or prevent CAUTIs. However, due to overuse of antibiotics for urinary tract infections, antibiotic resistance among bacterial uropathogens has increased in the past decades. Nowadays, antibiotic resistance is a serious global health threat, causing 1.2 million deaths worldwide in 2019 (10). One of the most effective strategies to minimize antibiotic resistance is to reduce the use of antibiotics (26). Especially with more patients using catheters each year (4), alternative treatment options for CAUTIs should be considered. In this study we investigated whether BI with tap water could reduce the need for antibiotic treatment for CAUTIs without symptoms of tissue invasion.

To the best of our knowledge, this is the first study that evaluated BI with tap water as a treatment and prevention for CAUTIs in NLUTD and non-NLUTD patients. Our study showed that nearly half of the patients experienced no CAUTI symptoms during the last three months of BI, while the majority of these patients suffered from recurrent CAUTIs before starting with this treatment. More importantly, only half of all self-reported CAUTIs were treated with antibiotics. This may indicate that CAUTIs have successfully been treated with BI and may lead to significantly less antibiotic use. However, due to the design of our study, we should mention that no firm conclusions can yet be drawn based on these initial data. On the other hand, the findings of our study are promising and provide a basis for future work that is important to counter the aforementioned global health threat of antibiotic resistance. It has been recommended to control and minimize antibiotic use on a global scale including low-income countries to overcome antibiotic resistance (10). Tap water is readily available worldwide, making BI easy to use and implement as a treatment for CAUTIs (15, 16). However, not in every country is tap water of high quality and constantly microbiologically monitored. In these countries, bottled water could be considered to irrigate the urinary bladder. Both tap and bottled water are inexpensive and easily available for patients compared to antibiotics or sterile saline for BI. Moreover, a potential additional advantage of irrigation with tap water is its hypo-osmolarity. The exposure of a hypo-osmolar medium to microorganisms, such as E. coli, results in loss of viability and lysis of the microbes (19). This could theoretically reduce the risk of bacteriuria and CAUTIs.

To date, very little research has been done on the efficacy of bladder irrigation in the treatment of CAUTIs. A Cochrane review by Shepherd et al. reported inconclusive evidence on the efficacy of BI in the prevention of CAUTIs in indwelling urinary catheter (IUC) patients (12). They included seven studies that compared different BI solutions and regimens for the prevention of CAUTIs and catheter blockage. All of these studies contained a high risk of bias, were generally underpowered, and were of poor methodological quality (12). Only four studies compared saline or acidic solution with no washout regimen. Thus, there is a lack of reliable evidence on the safety and efficacy of BI in IUC patients. Furthermore, this Cochrane review only investigated BI as a strategy for the prevention of CAUTIs and not as a treatment for CAUTIs. In addition, a prospective study by Husmann reported that daily BI with 240 mL of saline was associated with a significant decrease in recurrent bladder calculi and symptomatic urinary tract infections in spina bifida patients with augmented bladders (27).

Furthermore, we found that both NLUTD and non-NLUTD patients were generally satisfied with BI with tap water and would recommend the treatment to other patients. Several predictors for reporting higher treatment satisfaction outcomes were also identified, including being female and having fewer self-reported CAUTIs. The Cochrane review by Shepherd et al. reported that no previous studies have addressed BI acceptability measures, such as treatment satisfaction or ease of use. While treatment satisfaction is increasingly recognized as an important measure. Increased treatment satisfaction is associated with higher compliance and persistence, and with lower regimen complexity or treatment burden (28). We could hypothesize that the high treatment satisfaction in our study has a positive effect on patient adherence and persistence with BI, which is ultimately important for long-term treatment efficacy.

Patients with NLUTD reported more often an improvement in their condition after starting with BI compared to patients without NLUTD. There was an improvement on the PGI-I scale of 85.4% of NLUTD patients and 65.5% of non-NLUTD patients. This might be explained by the fact that non-NLUTD patients are more likely to compare their current situation with their situation without any bladder symptoms, making them less likely to notice and be satisfied with minimal improvements. While neurogenic patients adapt more easily to a new situation or treatment and will notice small improvements easier. Differences between NLUTD and non-NLUTD patients have been described previously in a study of antimuscarinic use, in which neurogenic patients also showed better persistence than patients with idiopathic overactive bladder (29). From this perspective, the difference could also be explained by the fact that in NLUTD patients, complications are more likely to have serious consequences, such as complicated urinary tract infections or high-pressure bladders during filling. Hence, NLUTD patients are more likely to perceive less fear of complications and, therefore, experience more improvement once they have fewer CAUTI symptoms compared to non-NLUTD patients.

In the Netherlands, BI appears to be more widely used in current practice of CAUTI treatment than previously anticipated. This result is surprising because BI is not described in the professional guidelines of the EAU or AUA and there exists no information on the current use of BI in the prevention and treatment of CAUTIs in other countries. Urologists and urology residents from 32 of 82 hospitals in the Netherlands responded to our survey, of which a small majority used BI as a treatment for CAUTIs. Most of the surveyed urologists and urology residents used sterile saline for BI instead of tap water. However, our results showed that BI with tap water is effective and results in less antibiotic use. Moreover, a randomized controlled trial showed that tap water is safe, cost-effective and more patient-friendly compared to sterile saline for daily irrigation of continent catheterizable ileal pouches (30). These patients had fewer nitrite-positive days, indicating that tap water decreased the incidence of bacteriuria, whether symptomatic or asymptomatic. In line with our findings, the patients included in this study were overall very satisfied with tap water irrigation. Therefore, we believe that tap or drinking water should be used more often for urinary bladder irrigation.

Strengths of our cross-sectional study include the high response rate of 70.0% and the use of a standardized bladder irrigation protocol. This enables an adequate reflection of our bladder irrigation regime in both NLUTD and non-NLUTD patients with IUCsor performing CIC in the Erasmus MC. To our knowledge, this is the first study that evaluated BI with tap water as a treatment for CAUTIs, and in which treatment satisfaction is included. Treatment satisfaction is important for the adherence and long-term efficacy of BI. The findings of our study are promising and encourage further research. Randomized controlled trials are needed to confirm the efficacy and safety of BI with tap water. This will be the next step toward utilizing BI with tap water in the prevention and treatment of CAUTIs.

In addition, our clinician survey is the first to provide insight into the current use of BI in the prevention and treatment of CAUTIs. We think BI could be mentioned in the professional guidelines as an alternative option to antibiotic treatment. This latter statement could be strengthened if future trials confirm the efficacy and safety of BI with tap water. This would enable other countries to implement BI in their clinical practice and ultimately reduce the use of antibiotics.

Our study also has some limitations. First, the cross-sectional and patient-reported nature of this study cannot provide robust evidence for the efficacy and safety of tap water irrigation, as no comparisons between CAUTI treatment modalities or interpatient changes in CAUTI incidence were conducted. Moreover, within our study design we did not evaluate changes in urine cultures before and after BI. Although this is not a prospective longitudinal study or randomized controlled trial, it is the best level of evidence so far and a first step towards a prospective study. Second, our findings are at risk to be biased towards better outcomes, because patients who discontinued BI were not included in this study. One could hypothesize that these patients were not satisfied or did not experience any improvement with BI. For this reason, we are currently conducting a prospective study to provide more solid evidence.

In addition, in our clinician survey there might be a response bias and we did not include continence nurses, nursing home physicians or general practitioners. While these clinicians often care for patients with urinary catheters.

## 5 Conclusions

Our results provide evidence supporting the efficacy of bladder irrigation with tap water in the treatment of CAUTIs and reducing the use of antibiotics. Both NLUTD and non-NLUTD patients are overall satisfied and experience improvement in their condition with this treatment. Therefore, BI with tap water might be considered a promising, easy to implement and more cost-effective alternative to antibiotics for the treatment of CAUTIs without symptoms of tissue invasion. To our surprise, a small majority of the Dutch clinicians surveyed use BI for the prevention and treatment of CAUTIs, although it is not described in professional guidelines. On the other hand, irrigation with tap water is still not widely used. Future randomized studies are needed in order to confirm the efficacy and safety of BI with tap water.

## Data availability statement

The raw data supporting the conclusions of this article will be made available by the authors, without undue reservation.

## Ethics statement

The studies involving human participants were reviewed and approved by MEC-2021-0855. The patients/participants provided their informed consent to participate in this study.

## Author contributions

All authors declare to the study conception and design. Material preparation, data collection and analysis were performed by FV, SD, RC and JB. The first draft of the manuscript was written by FV and all authors commented on previous versions of the manuscript. All authors read and approved the final manuscript. All authors contributed to the article and approved the submitted version.
